# Structure and Interactions of a Dimeric Variant of sHIP, a Novel Virulence Determinant of *Streptococcus pyogenes*

**DOI:** 10.3389/fmicb.2016.00095

**Published:** 2016-02-05

**Authors:** Carl Diehl, Magdalena Wisniewska, Inga-Maria Frick, Werner Streicher, Lars Björck, Johan Malmström, Mats Wikström

**Affiliations:** ^1^Protein Function and Interactions Group, Faculty of Health and Medical Sciences, The Novo Nordisk Foundation Center for Protein Research, University of CopenhagenCopenhagen, Denmark; ^2^SARomics BiostructuresLund, Sweden; ^3^Malopolska Centre of BiotechnologyKrakow, Poland; ^4^Division of Infection Medicine, Department of Clinical Sciences, Lund UniversityLund, Sweden; ^5^Novozymes A/SBagsvaerd, Denmark

**Keywords:** proteomics, structural biology, virulence factors, protein-protein interactions, host-pathogen relationship

## Abstract

*Streptococcus pyogenes* is one of the most significant bacterial pathogens in the human population mostly causing superficial and uncomplicated infections (pharyngitis and impetigo) but also invasive and life-threatening disease. We have previously identified a virulence determinant, protein sHIP, which is secreted at higher levels by an invasive compared to a non-invasive strain of *S. pyogenes*. The present work presents a further characterization of the structural and functional properties of this bacterial protein. Biophysical and structural studies have shown that protein sHIP forms stable tetramers both in the crystal and in solution. The tetramers are composed of four helix-loop-helix motifs with the loop regions connecting the helices displaying a high degree of flexibility. Owing to interactions at the tetramer interface, the observed tetramer can be described as a dimer of dimers. We identified three residues at the tetramer interface (Leu84, Leu88, Tyr95), which due to largely non-polar side-chains, could be important determinants for protein oligomerization. Based on these observations, we produced a sHIP variant in which these residues were mutated to alanines. Biophysical experiments clearly indicated that the sHIP mutant appear only as dimers in solution confirming the importance of the interfacial residues for protein oligomerisation. Furthermore, we could show that the sHIP mutant interacts with intact histidine-rich glycoprotein (HRG) and the histidine-rich repeats in HRG, and inhibits their antibacterial activity to the same or even higher extent as compared to the wild type protein sHIP. We determined the crystal structure of the sHIP mutant, which, as a result of the high quality of the data, allowed us to improve the existing structural model of the protein. Finally, by employing NMR spectroscopy in solution, we generated a model for the complex between the sHIP mutant and an HRG-derived heparin-binding peptide, providing further molecular details into the interactions involving protein sHIP.

## Introduction

*Streptococcus pyogenes*, also known as group A streptococci (GAS) is a significant human pathogen that infects and colonizes the skin and the upper respiratory tract where it causes relatively mild clinical conditions such as impetigo and pharyngitis. Some infections caused by invasive strains, such as the AP1 strain of the M1 serotype, can lead to severe and potentially life-threatening diseases such as necrotizing fasciitis and streptococcal toxic shock syndrome (STSS), whereas acute rheumatic fever and glomerulonephritis are sequelae to acute *S. pyogenes* infections. *S*. *pyogenes* causes an estimated 700 million cases of mild and non-invasive infections each year, of which ~650,000 progress to severe invasive infections with a mortality of at least 25% (Carapetis et al., 2005; Ralph and Carapetis, 2013). *S. pyogenes* produces a number of proteins that enable the bacterium to attach to host tissues, evade the immune response, and spread by penetrating host tissue layers. These virulence factors are predominantly secreted or surface associated proteins, and they include the family of M proteins (Lancefield, 1962; Swanson et al., 1969; Phillips et al., 1981), fibronectin-binding proteins (Talay et al., 1994; Kreikemeyer et al., 1995; Jaffe et al., 1996; Courtney et al., 1999; Rocha and Fischetti, 1999; Terao et al., 2001), super-antigenic exotoxins (Stevens et al., 1989; Abe et al., 1991; Tomai et al., 1992; Mollick et al., 1993; Norrby-Teglund et al., 1994), and the secreted streptococcal inhibitor of complement referred to as protein SIC (Åkesson et al., 1996; Fernie-King et al., 2002; Frick et al., 2003).

In a recent study, we quantitatively analyzed and compared *S. pyogenes* proteins in the growth medium of a strain that is virulent to mice (AP1), with a non-virulent strain (SF370). We found that one protein in particular was present at significantly higher levels in the stationary growth medium from the virulent strain. The amount of sHIP in the medium fraction is similar to the secreted mitogenic exotoxin SmeZ (Kamezawa et al., 1997), and shows an overall abundance profile resembling the profile observed for the surface associated proteins H and M1 (Lancefield, 1962; Åkesson et al., 1990; Wisniewska et al., 2014). Through the use of affinity pull-down mass spectrometry analysis of human plasma, we could demonstrate that the new bacterial protein interacts with the antimicrobial human protein histidine-rich glycoprotein (HRG), and the name sHIP (streptococcal Histidine-rich glycoprotein Interacting Protein) was therefore introduced (Wisniewska et al., 2014). HRG is an abundant plasma glycoprotein of approximately 60 kDa that interacts with several other protein ligands such as tropomyosin, heparin, plasminogen, plasmin, fibrinogen, and IgG (Jones et al., 2005). HRG has been shown to exhibit broad antimicrobial potency (Rydengård et al., 2007), including activity against *S. pyogenes* (Shannon et al., 2010). It was shown that sHIP binds both intact HRG and HRG-derived peptides (peptides containing consensus heparin-binding sequences) with high affinity. Moreover, the antibacterial activity of HRG is blocked by protein sHIP, which represents a new mechanism that can contribute to the virulence of AP1 bacteria. In addition, we could show that patients with severe *S. pyogenes* infection, in contrast to patients with superficial and uncomplicated infections, are more prone to develop antibodies against sHIP, which suggests that sHIP represents a novel virulence determinant (Wisniewska et al., 2014). Furthermore, the determination of the three-dimensional structure of sHIP, showed that it has a tetrameric organization composed of four helix-loop-helix motifs. A similar structural unit can be found in the adhesion factor FadA from *Fusobacterium nucleatum*. However, the two proteins differ significantly in their respective oligomeric organization. In FadA, the helix-loop-helix motifs form elongated fibers whereas the sHIP monomers are organized into a compact tetrameric structure. In order to understand the molecular prerequisites for the observed interaction between sHIP and HRG, we have performed extensive crystallization trials but not been able to obtain any diffracting crystals of complexes between sHIP and HRG or any HRG derived peptides. Due to the size of the tetrameric sHIP, NMR experiments in solution have proven to be very challenging and would benefit from access to a sHIP variant with lower molecular weight and retained activity. We have previously made the observation that the tetrameric structure of sHIP can also be described as a dimer of dimers, and postulated that three residues in the dimer-dimer interface could represent important determinants for the stabilization of the tetrameric form of sHIP. In this study, we have mutated these three interfacial residues and thereby been able to produce a stable dimeric variant of sHIP that was shown to be active both in biophysical binding experiments and an antimicrobial assay. The generation of stable dimers enabled further characterization of the interaction between sHIP and a peptide from HRG through NMR experiments, providing the first molecular details of an interaction involving this novel virulence determinant.

## Materials and methods

### Mutagenesis

The DNA sequence corresponding to residues Lys3-Met98 in the *S. pyogenes* protein sHIP (UniprotID: Q99XU0) was expressed as described previously and denoted sHIPwt (Wisniewska et al., 2014). A protein sHIP variant, a quadruple mutant named sHIPqp, in which Leu84, Leu88, Tyr95 were mutated to alanine, and Cys65 was mutated to serine, was constructed using the Quikchange XL site-directed mutagenesis kit (Agilent) following the manufacturer's instructions. We have previously observed that the wildtype protein can form larger aggregates through the exposed Cys65 residue in the absence of a reducing agent. The mutation Cys65Ser was introduced in order to avoid the need to use a reducing agent in subsequent experiments. As previously noted, this mutation has no effect on the structure and function of sHIP (Wisniewska et al., 2014). We are therefore using the Cys65Ser mutant in all studies involving both the wildtype sHIP and in the sHIP variant. All mutations were verified by DNA sequence analysis. The resulting expression construct contained a His-tag and a TEV protease cleavage site preceding the protein sequence of interest.

### Protein expression and purification

The protein sHIP variants sHIPwt and sHIPqp were expressed in *E. coli* strain Rosetta BL21 (DE3) and purified in two steps using standard immobilized metal ion affinity chromatography (IMAC) followed by proteolytic removal of the His-tag, and reverse phase chromatography (RPC), as described previously (Wisniewska et al., 2014). For the NMR studies, protein was produced using minimal medium for cell growth in order to introduce the stable isotopes ^15^N and ^13^C enabling triple-resonance NMR experiments (Neidhardt et al., 1974). The His-tag was removed by treatment with tobacco etch virus (TEV) protease giving the following amino acid sequences for the wildtype protein sHIP, sHIPwt:

SMKQDQLIVEKMEQTYEAFSPKLANLIEALDAFKEHYEEYATLRNFYSSDEWFRLANQPWDDIPCGVLSEDLLFDMIGDHNQLLADILDLAPIMYKHM,

and the quadruple mutant protein sHIP, sHIPqp:

SMKQDQLIVEKMEQTYEAFSPKLANLIEALDAFKEHYEEYATLRNFYSSDEWFRLANQPWDDIPSGVLSEDLLFDMIGDHNQLAADIADLAPIMAKHM, respectively.

The purity and mono-dispersity of the recombinant proteins were verified by SDS-PAGE electrophoresis and mass spectrometry.

### Crystallization, X-ray data collection, and structure determination

Crystallization of the protein sHIP variant was carried out using the sitting drop vapor diffusion method at 18°C. The best crystals were obtained from 20% PEG 6000, 0.2 M calcium chloride and 0.1 M Tris, pH 8.0. Prior to plunge freezing, the crystals were soaked for approximately 30 s in a drop of a reservoir solution containing 20% v/v ethylene glycol as cryo-protectant. The crystals belonged to the space group P22121 and contained one monomer per asymmetric unit. The highest resolution data set of 180 frames (1° oscillation range) was collected from an orthorhombic crystal at Max II lab beamline I911-2, Lund, Sweden, using a MAR CCD detector. Data were indexed and integrated using iMOSFLM (Battye et al., 2011) and scaled using SCALA (Evans, 2006) from the CCP4 suite of programs. The structure was solved by molecular replacement using wild type sHIP as the search model (PDB ID: 4MER), and refined using REFMAC (Murshudov et al., 1997). Refinement rounds were complemented with manual rebuilding using COOT (Emsley et al., 2010). Water molecules were automatically inserted using Arp/wArp and visually inspected. For further details on data processing and refinement statistics, see Table 1. Geometry of the model was analyzed with Molprobity (Chen et al., 2010). Molecular graphics images were generated using the program Pymol. The atomic coordinates and structure factors for the sHIP mutant (sHIPqp) crystal structure have been deposited with the protein data bank (PDB ID: 4PZ1).

**Table 1:** **Crystallographic data collection and refinement statistics**.

**Protein**	**sHIPqp mutant**
**DATA COLLECTION**	
Beamline	I911-2
Wavelength (Å)	1.038
Space group	P*22121*
Cell dimensions	
*a, b, c* (Å)	29.37 33.28 89.58
*α, β, γ* (°)	90 90 90
Resolution (Å)	19.76–1.73 (1.82–1.73)
R__meas_(%)	6.4 (14.6)
I/sigI	19.9 (10.3)
Completeness (%)	98.8 (97.8)
Multiplicity	6.4 (6.2)
**REFINEMENT**	
Resolution (Å)^a^	19.76–1.73 (1.77–1.73)
No. reflections	9077 (657)
R_work_/R_free_	18.9/21.1 (20.3/38)
**NO. ATOMS**	
Protein	792
Chloride	2
Calcium	1
Water	78
***B*****-FACTORS (Å^2^)**	
Protein	14.8
Water	20.56
**RMSD STEREOCHEMISTRY^b^**	
Bond lengths (Å)	0.011
Bond angles (°)	1.295
**RAMACHANDRAN PLOT (%)**	
Most favored regions	100

a *Values for the highest resolution shell values are indicated in parentheses*.

b *RMSD, root mean square deviation*.

*alpha, beta, gamma refers to the unit cell axes dimensions, while alpha, beta, and gamma in symbol refers to the inclination angles of the axes in the unit cell. The space group number P22121 refers to the description of the symmetry of the crystal*.

### Analytical ultracentrifugation

Sedimentation velocity experiments were performed at 20°C and 50000 rpm using a Beckman XL-I instrument. Samples containing either sHIPwt or sHIPqp were in 20 mM MES buffer (2-(N-morpholino) ethanesulfonic acid), pH 5.5. The data were analyzed with SEDFIT using a continuous c (S) distribution (Schuck, 2000). HYDROPRO (Ortega et al., 2011) was used to calculate the theoretical sedimentation coefficient using the PDB IDs 4MER (sHIPwt) and 4PZ1 (sHIPqp), respectively.

### Synthetic peptide

The sequence for the HRG peptide used in the study referred to as HRGsingle, corresponding to a single heparin-binding motif, have the amino acid sequence GHHPHG. The peptide was synthesized by Biosyntan GmbH (Berlin, Germany) and purity and molecular mass were confirmed by MALDI-TOF MS.

### Antimicrobial assay

The *S. pyogenes* strain AP1 (40/58), was from the World Health Organization Collaborating Centre for Reference and Research on Streptococci, Prague, Czech Republic. The bacteria were cultivated in THY (Todd-Hewitt broth (Difco) supplemented with 0.2% yeast extract (Oxoid) at 37°C and 5% CO_2_ until reaching mid-log phase (OD 620 nm approximately 0.4). The bacterial cells were washed and resuspended in 10 mM Tris-HCl, pH 7.5, containing 5 mM glucose, to a concentration of 2 × 10^9^ cfu (colony forming unit)/ml. Subsequently, the bacteria were diluted to a concentration of 2 × 10^6^ cfu/ml in 10 mM MES buffer, pH 5.5, containing 5 mM glucose. Fifty microliters of the bacterial solution was incubated with recombinant His-tagged HRG (Creative Biomart) at a concentration of 0.45 μM together with various concentrations of protein sHIPwt or protein sHIPqp for 40 min at 37°C. Serial dilutions of the incubation mixtures were plated on TH agar, plates were incubated over night at 37°C and the number of cfu's were determined.

### Isothermal titration calorimetry (ITC)

ITC experiments were performed using a VP-ITC200 instrument (GE Healthcare). The samples were extensively dialyzed against 20 mM MES buffer, pH 5.5. 0.5 mM HRGsingle peptide was titrated into 0.05 mM of sHIPwt, or sHIPqp respectively. All experiments were performed at 25°C and run until saturation was achieved. The data were fitted using a model describing one independent binding site using the software provided by the manufacturer (Wiseman et al., 1989).

### NMR experiments

Protein samples were prepared in 20 mM MES pH 5.5 with 7% (v/v) D2O for the spectrometer lock and 0.02% (w/v) NaN_3_ to prevent bacterial growth in the samples. All NMR experiments were performed at 25°C. For the studies of the sHIPqp/HRGsingle peptide complex, the sample contained 1.0 mM of double-labeled (^15^N, ^13^C) protein sHIPqp and 1.1 mM of the HRGsingle peptide. The backbone and side-chain resonance assignment experiments were performed at 800 MHz proton resonance frequency, whereas the experiments in order to acquire Nuclear Overhauser Enhancement (NOE) distance restraints were acquired at 900 MHz proton resonance frequency. The assignment of the signals from sHIPqp and the HRGsingle peptide, and collection of NOE restraints were performed by using a combination of standard 2D and 3D triple-resonance experiments including HNCA (Clore et al., 1990), HNCOCA (Bax and Ikura, 1991), HNCACB (Wittekind and Mueller, 1993), CBCACONH (Grzesiek and Bax, 1992), HCCONH (Montelione et al., 1992), CCONH (Montelione et al., 1992), ^15^N-NOESY-HSQC (Marion et al., 1989), ^13^C-NOESY-HSQC (separate for aliphatic and aromatic signals; Marion et al., 1989), 2D double-filtered NOESY (Ikura et al., 1992), and 2D single-filtered NOESY (Ikura et al., 1992). In addition, 2D NOESY and TOCSY experiments were acquired for the HRGsingle peptide. Spectra were processed with NMRpipe (Delaglio et al., 1995), applying zero-filling and linear prediction in the indirect dimensions and solvent filter and polynomial baseline correction in the direct dimension. CCPNMR (Vranken et al., 2005) was used for visualization of spectra, resonance connectivity analysis, and distance restraints (NOE) assignments. Structure calculations were performed using an iterative procedure within the ARIA/CNS suite of programs (Brunger et al., 1998). The assessment of the quality of the NMR generated structural models, were performed using the Protein Structure Validation Suite (PSVS, **Table 3**; Bhattacharya et al., 2007). Molecular graphics images were generated using the program Pymol (**Figure 5**).

## Results

### The sHIPqp mutant forms stable dimers of two helix-loop-helix motifs

**Figure 1:**
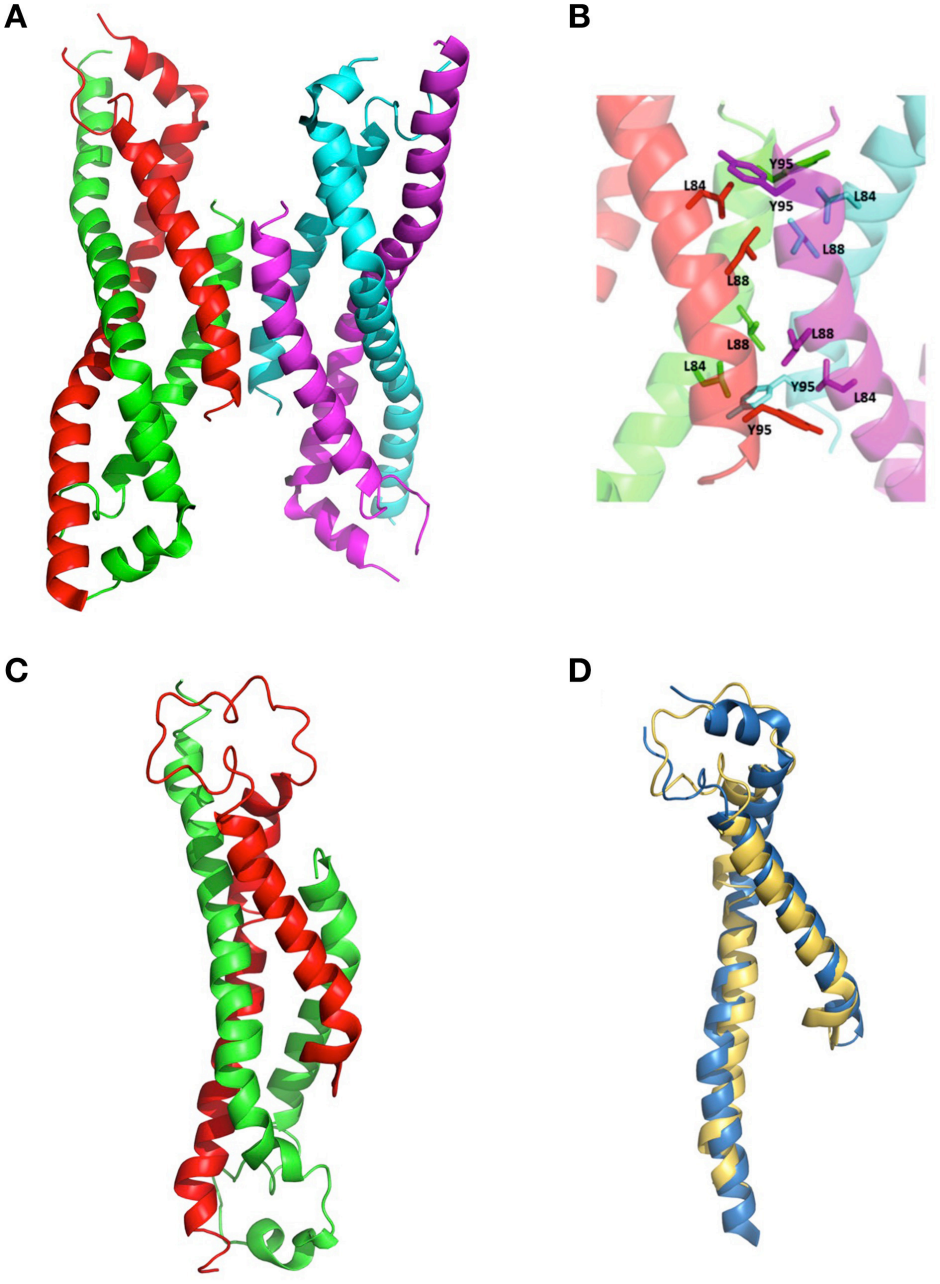
**Three-dimensional structure of sHIP**. **(A)** The sHIPwt homotetramer formed by chain A (green), chain B (red), chain C (magenta), and chain D (cyan) using the PDB ID 4MER. **(B)** Molecular interface showing the residues in contact at the interface of the two dimers of the sHIPwt tetramer (PDB ID: 4MER). **(C)** The sHIPqp dimer formed by chain A (green), and chain B (red) (PDB ID: 4PZ1). **(D)** Three-dimensional alignment of the structures of the helix-loop-helix monomers of wildtype sHIP (blue) and the sHIPqp mutant (yellow).

**Figure 2:**
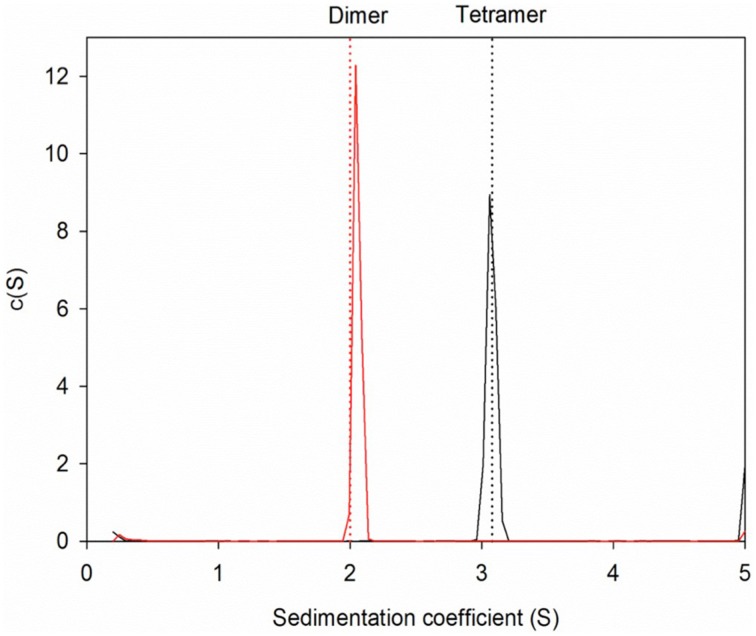
**Sedimentation velocity experiments for protein sHIP**. The dotted lines indicate the theoretical sedimentation coefficient for a tetramer and a dimer respectively calculated using HYDROPRO and the structural coordinates for protein sHIPwt (PDB ID: 4MER) and protein sHIPqp (PDB ID: 4PZ1). The wild-type sHIPwt is shown in black, and the sHIPqp mutant in red respectively.

The wildtype sHIP is composed of tetramers that can be described as the dimer of two dimers each harboring two helix-loop-helix motifs (Figure 1A). The hypothesis was that the three non-polar residues at the tetramer interface (Leu84, Leu88, Tyr95) represent important determinants for the protein oligomerization (Figure 1B), and these residues were therefore mutated to alanines in the sHIPqp mutant protein. The orthorhombic crystals of the sHIPqp mutant contain one monomer in the asymmetric unit, with a solvent content of 36%. The crystal packing shows the formation of a dimer with a total buried surface area of 6070 Å^2^ as calculated by PISA server (Krissinel and Henrick, 2007). The final model comprises residues 3-98, corresponding to the entire sHIPqp molecule (Figure 1C). The structure is very well defined, with an average B factor of 12.2 Å^2^ (10.8 Å^2^ and 13.6 Å^2^ for main chain and side chains, respectively). Final electron density maps are of very high quality including the loop region, which was partially not visible in sHIP wild type (Figure 1D). All non-glycine residues exhibit main-chain angles in the favored regions of the Ramachandran plot as defined by the use of the program Molprobity (Chen et al., 2010; Table 1). To provide a quantitative analysis of the oligomeric states of the sHIP variants, analytical ultracentrifugation was employed on sHIPwt and the sHIPqp mutant. These experiments clearly show that the wildtype sHIP (sHIPwt) appear as tetramers in solution whereas the sHIP mutant (sHIPqp) exist as dimers (Figure 2). The largest differences in the monomeric helix-loop-helix motif between the two sHIP variants are observed in the loop region connecting the two helices. In the sHIPqp mutant, all residues, including side-chains, are very well defined, while in sHIPwt, some residues could not be defined due to the absence of interpretable electron density maps in this region (Figure 1D).

### The sHIPqp mutant inhibits the antibacterial activity of HRG

**Figure 3:**
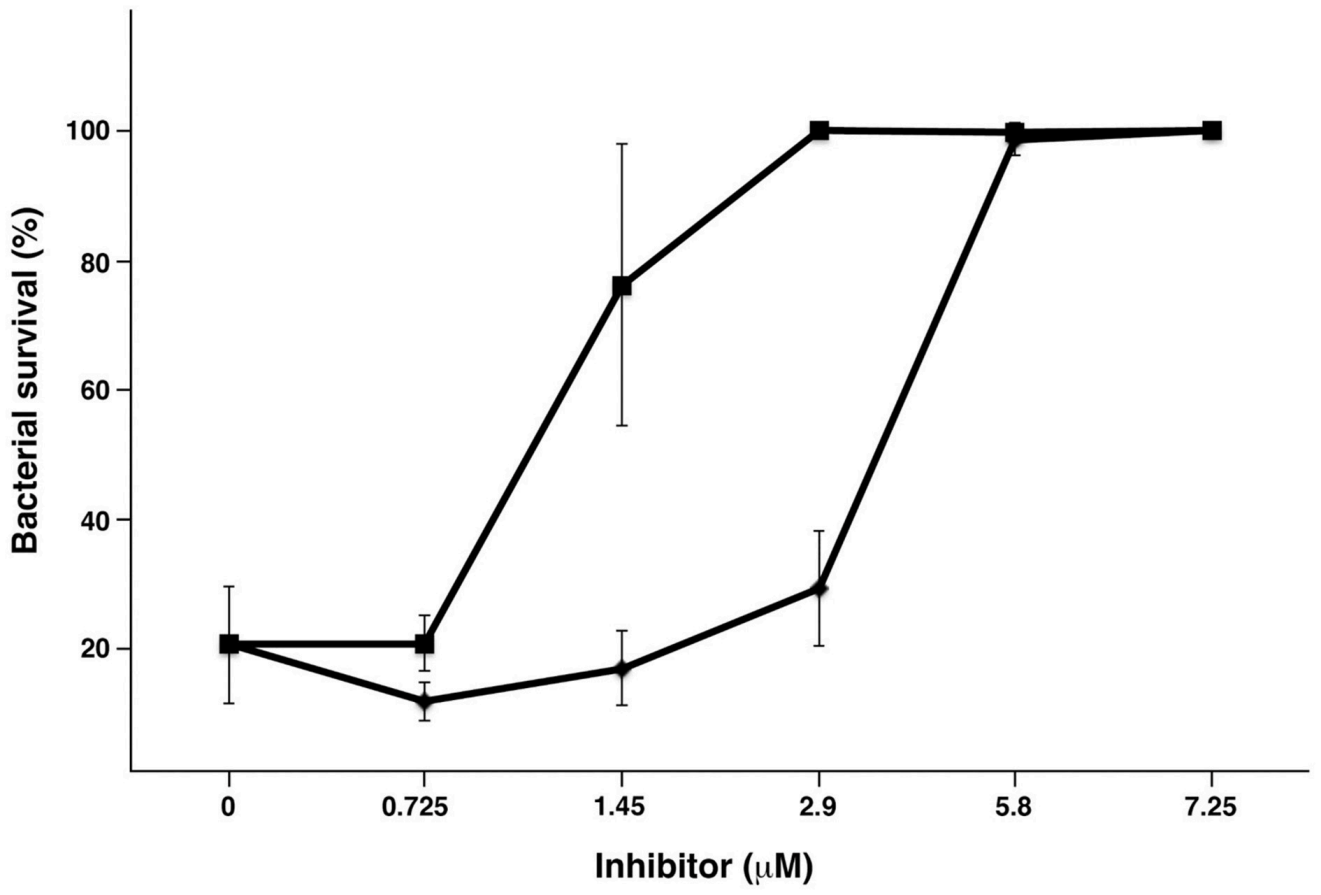
**Protein sHIPqp mutant inhibits the bactericidal activity of HRG more efficiently than protein sHIP wildtype**. The bactericidal effect of HRG at 0.45 μM against *S. pyogenes* AP1 bacteria (2 × 10^6^ cfu/ml) was inhibited with sHIP wildtype (♦), and sHIPqp mutant (■) at indicated concentrations. Experiments were repeated at least three times and mean values ± SD are shown.

It is known that HRG has antibacterial activity including activity against *S. pyogenes* (Shannon et al., 2010), and when challenged by HRG, sHIP has been shown to rescue *S. pyogenes* bacteria (Wisniewska et al., 2014). To examine whether a sHIP mutant has the same ability, we performed an antimicrobial assay comparing sHIPwt and sHIPqp. As shown in Figure 3, sHIPqp inhibits the bactericidal activity of HRG to the same or even higher extent that wildtype sHIP indicating that the dimeric mutant sHIPqp retains its inhibitory capacity.

### sHIP binds a single heparin-binding motif in HRG

**Figure 4:**
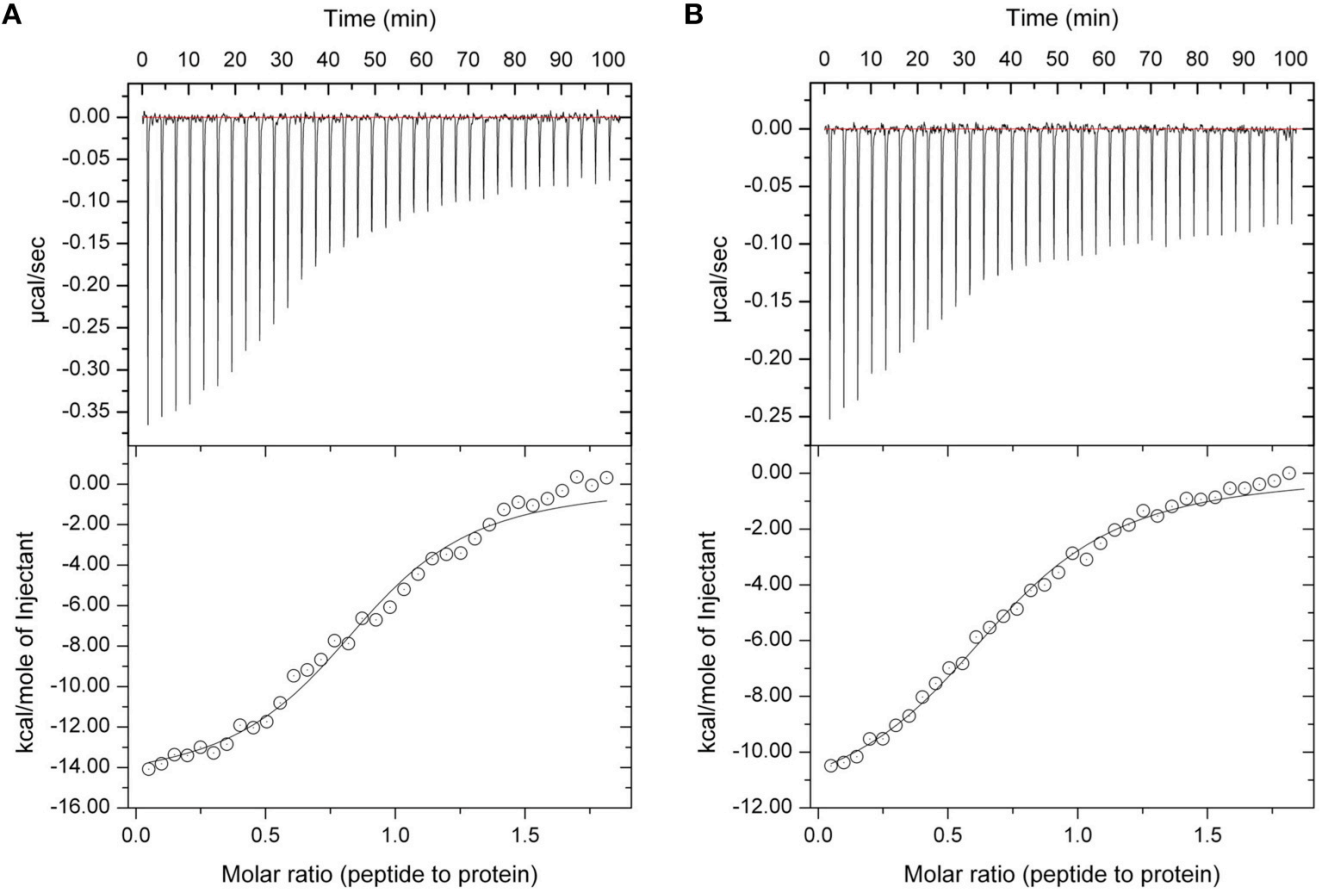
**Isothermal titration Calorimetry (ITC) experiments showing the binding between sHIP and the HRGsingle peptide. (A)** sHIPwt and HRGsingle. **(B)** sHIPqp and HRGsingle. The raw data of the experiments are presented on the top panel. The area underneath each injection peak is equal to the total heat released for that injection. When this integrated heat is plotted against the molar ratio of titrant added to the peptide solution in the cell, a complete binding isotherm for the interaction is obtained (bottom panel). A model for either one binding site was used to fit the data. The solid line is the calculated curve using the best-fit parameters.

In our previous studies we have shown that protein sHIP binds to both HRG and HRG-derived peptides with nanomolar to micromolar affinity, and that the histidine-rich region is important for the interaction (Wisniewska et al., 2014). To further dissect the molecular details in the interaction, we investigated whether the sHIP variants are also able to bind a single heparin-binding motif (GHHPHG) in HRG (denoted as HRGsingle). Isothermal titration calorimetry (ITC) experiments showed that both sHIPwt and sHIPqp binds HRGsingle with similar micro-molar affinities, clearly indicating that the heparin-binding motif represents an important recognition motif in HRG for the interaction with protein sHIP (Figure 4, Table 2).

**Table 2:** **Summary of thermodynamic parameters from ITC experiments of the binding of the two sHIP variants sHIPwt and sHIPqp to the HRGsingle peptide respectively**.

**Complex**	**N (sites)**	**K_d_ (μM)**	**ΔH (kcal/mol)**
sHIPwt/HRGsingle	0.90 ± 0.02	1.9 ± 0.2	−15.1 ± 0.4
sHIPqp/HRGsingle	0.79 ± 0.03	2.8 ± 0.3	−12.2 ± 0.3

### The HRG-binding site in sHIP involves both helix-loop-helix motifs of the dimer

The wildtype protein sHIP is a tetrameric protein with a molecular weight of 46 kDa complicating NMR experiments in solution due to broad and overlapping resonances. The creation of the mutant dimeric form of sHIP, sHIPqp, and through the verification of its functional properties, we now have access to a smaller, yet biologically relevant system with a molecular weight of 23kDa, which enabled detailed NMR studies in solution.

**Figure 5:**
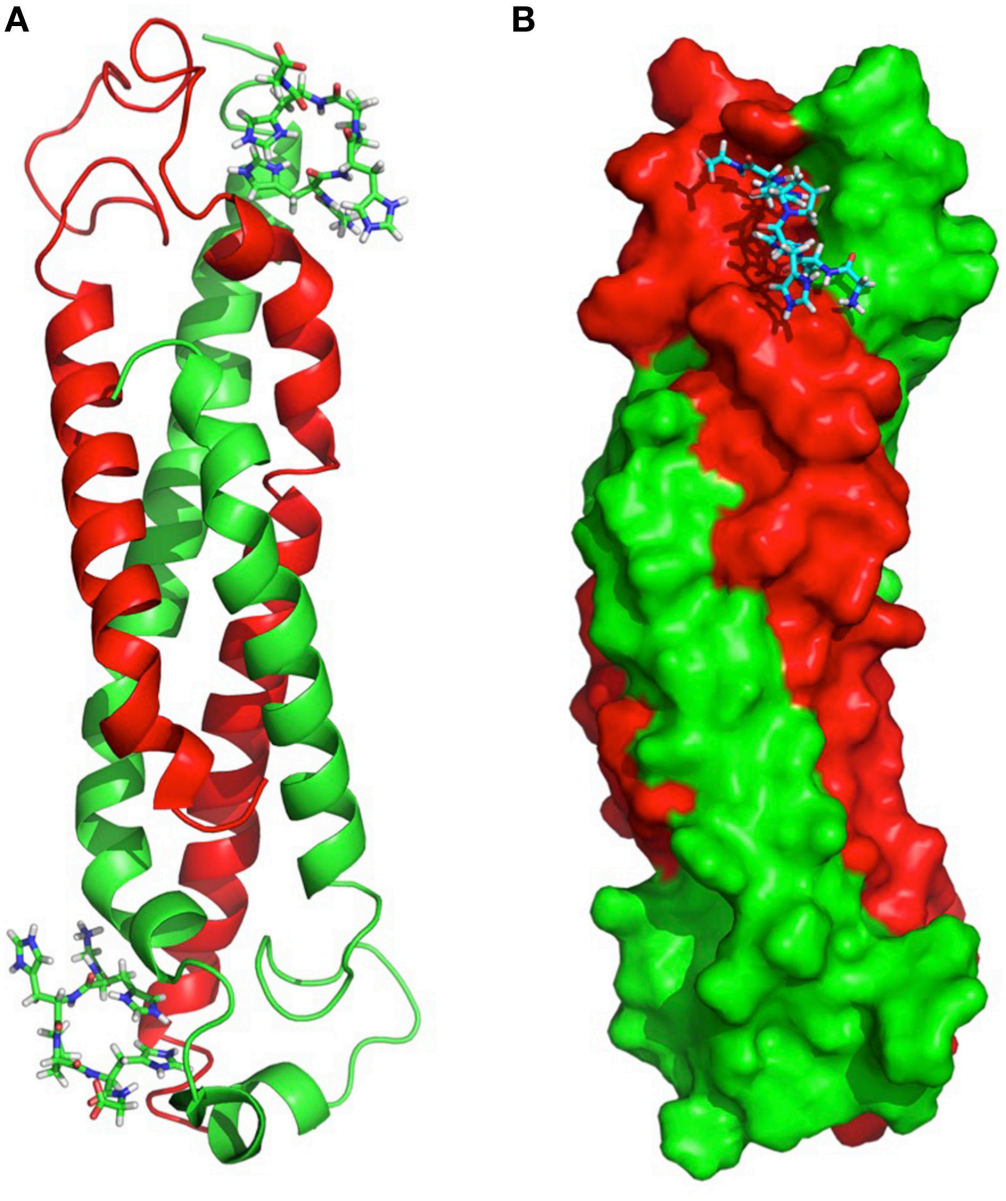
**Molecular model of the complex between sHIPqp and the HRGsingle peptide. (A)** sHIPqp is shown in a ribbon representation with chain A in green and chain B in red. The HRGsingle peptide is shown in a stick representation. **(B)** sHIPqp is shown in a space filling representation with chain A in green and chain B in red. The HRGsingle peptide is shown in a stick representation.

A molecular model was created with a combination of the structure of sHIPqp (PDB ID: 4PZ1), and distance restraints (NOEs) extracted from the NMR experiments of the complex between sHIPqp and the HRGsingle peptide. A total of 1714 NOEs were used as distance restraints in the structure calculations of which 321 are long-range restraints and 56 represent NOEs detected between sHIPqp and the HRGsingle peptide (Table 3). It should be noted that this model represent a preliminary description of the sHIPqp-HRGsingle complex that will need further refinement before considered a finalized complex structure. However, the assessment of the generated structure shows that it is of sufficient quality to represent a most relevant descriptive model for the complex (Table 3). The model shows that the HRGsingle peptide binds in a pocket created by residues from both monomeric helix-loop-helix motifs, suggesting that the dimeric form represents the smallest sHIP variant attainable with retained HRG-binding capacity. Each sHIP dimer binds two HRGsingle peptides in two identical sites (Figure 5A). The binding site includes the N-terminal part of helix 1 of one monomer (residues Asp5-Met12), and the end region of helix 1 of the second monomer (residues Ala41-Ala56). For the first monomer three residues are involved in NOE contacts with the HRGsingle peptide with primary contribution from Ile8. For the second monomer, thirteen residues give rise to detectable NOEs dominated by contacts involving Phe46, Glu51, and Arg54. Moreover, the binding site does not involve any residues in the proximity of the dimer-dimer interface in agreement with the observation that the mutant sHIPqp maintain its full inhibitory capacity of HRG in antimicrobial assays (Figure 3). The two external Glycine residues of the HRGsingle peptide, Gly-1 and Gly-6, are not involved in any interactions with sHIP. However, the core part of the peptide, (HHPH), forms multiple contacts with a binding pocket created by residues from both chains of the structural framework created by the sHIP dimer. In particular, residues His-2 and His-5 are positioned deeply in the binding pocket, a confirmation of the peptide backbone that is made possible due to the unique backbone properties of the connecting Pro-4 residue (Figure 5B).

**Table 3:** **NMR structural restraints and structure statistics of the sHIPqp/HRGsingle model**.

Summary of experimental restraints^a^	
NOE-based distance constraints:	
Total	1714
intra-residue [i = j]	403
sequential [| i - j | = 1]	467
medium range [1 < | i - j | < 5]	523
long range [| i - j | > 5]	321
sHIPqp/HRGsingle intermolecular NOEs	56
NOE constraints per restrained residue	17.0
Dihedral-angle constraints:	697
Total number of restricting constraints	2411
Total number of restricting constraints per restrained residue^b^	23.9
Restricting long-range constraints per restrained residue^b^	3.2
Structure Quality Factors–overall statistics	
^b^Procheck G-factor (phi/psi only)	−0.88
^b^Procheck G-factor (all dihedral angles)	−1.04
MolProbity clashscore	73.12
^c^Ramachandran plot (%)	
Most favored regions	70.1
Additionally allowed regions	28.3
Generously allowed regions	0.5
Disallowed regions	1.1

a *Analyzed for residues 2 to 98 (sHIPqp chain A), 102-198 (sHIPqp chain B), 301-306 (HRGsingle peptide 1), and 401-406 (HRGsingle peptide 2)*.

b *PROCHECK (Laskowski et al., 1993, 1996)*.

c *Ramachandran statistics calculated by PROCHECK (Laskowski et al., 1993, 1996)*.

## Discussion

Bacterial infections represent a major challenge to human health worldwide, a challenge increasing at an elevated pace due to the alarming upsurge of antibiotic resistance. In order to treat bacterial infections in the future, new therapeutic strategies need to be developed that will require a better understanding of the complex molecular interplay between bacterial pathogens and their human host. *S. pyogenes* represents one of the most significant bacterial pathogens in the human population. Using mass spectrometry based proteomics, we previously identified a novel protein and virulence factor from this bacterium (Wisniewska et al., 2014). In this study we have extended the structural and functional characterization of this protein to further our understanding of the interactions between sHIP and the human host.

The structure determination of the sHIPqp mutant showed that the fold of the sHIP mutant and the sHIP wild type monomers is essentially identical, displaying an elongated helix-loop-helix motif (Figure 1). However, the most significant difference is related to the oligomeric states of the two protein variants. For wildtype sHIP, four monomers assemble into a compact homotetramer, which can be described as a dimer of dimers due to the interactions at the dimer-dimer interface. Our previous studies showed that this interface consists of amino acids with largely non-polar side-chains, such as Leu84, Leu88, Tyr95, and Met98, which can be responsible for protein oligomerization. The mutation of these residues to alanine's resulted in a shift in quaternary structure from tetramers to dimers further evidenced by sedimentation velocity experiments that clearly show that the sHIPqp mutant exists as dimers in solution (Figure 2).

To gain further understanding of the interaction between sHIP and HRG, a molecular model was created for the complex between sHIPqp and a single heparin-binding motif of HRG (HRGsingle), a peptide shown to be active in binding to both sHIPwt and sHIPqp (Figure 4). The model shows that the HRGsingle peptide interacts with both monomers of the helix-loop-helix dimers, and that a proline situated in the middle of the peptide sequence allows two histidines, His-2 and His-5, to enter deeply into the binding pocket created by the two sHIP monomers (Figures 5A,B). However, even if both monomers contribute to contacts with the HRGsingle peptide, one of the monomers clearly dominates the overall contribution to the binding epitope for the sHIP dimer. Future studies should involve mutagenesis experiments of both sHIP and HRG peptides to further define the critical determinants for the interaction.

## Concluding remarks

Through structure-based protein engineering of a selected set of residues in the dimer-dimer interface of sHIP, we were able to generate a dimeric form of the protein with retained functional properties. The access to dimeric sHIP provides the opportunity to utilize NMR techniques in order to further elucidate interactions involving this protein. In conclusion, we have through a combination of protein engineering, biophysics, and structural biology methods, provided the first molecular details describing the interaction between the antimicrobial protein HRG and the novel virulence factor sHIP.

## Author contributions

CD and MagW performed the NMR and X-ray experiments, respectively. IF performed the antimicrobial assay, WS the AUC experiments, and JM the MS proteomics analysis. LB was providing the clinical data assessment. MW (corresponding author) supervised the project, performed the ITC experiments, and wrote the manuscript with input from all authors.

## Funding

This work was supported by the Swedish Research Council (projects 7480, 2008:3356, and 621-2012-3559), the Swedish Foundation for Strategic Research (grant FFL4), the Crafoord Foundation (grant 20100892), the Wallenberg Academy Fellow KAW (2012.0178), the European research council starting grant (ERC-2012-StG-309831), the Novo Nordisk Foundation grant NNF14CC0001, the European Commission under the Seventh Framework Program (FP7) contract Bio-NMR 261863 providing access to the Swedish NMR Centre, Gothenburg, Sweden, and the MAX laboratory, Lund, Sweden enabling access to the MAX laboratory (ID 20120014 and 20130189).

### Conflict of interest statement

The authors declare that the research was conducted in the absence of any commercial or financial relationships that could be construed as a potential conflict of interest.
